# “Long-term stability of stimulating spiral nerve cuff electrodes on human peripheral nerves”

**DOI:** 10.1186/s12984-017-0285-3

**Published:** 2017-07-11

**Authors:** Breanne P. Christie, Max Freeberg, William D. Memberg, Gilles J. C. Pinault, Harry A. Hoyen, Dustin J. Tyler, Ronald J. Triolo

**Affiliations:** 1 Case Western Reserve University, Cleveland, OH USA; 2 Department of Veterans’ Affairs, Louis Stokes Cleveland Medical Center, Cleveland, OH USA; 3 MetroHealth Medical Center, Cleveland, OH USA

**Keywords:** Functional electrical stimulation, Stability, Nerve cuff electrodes, Neural prostheses, Neural interfaces

## Abstract

**Background:**

Electrical stimulation of the peripheral nerves has been shown to be effective in restoring sensory and motor functions in the lower and upper extremities. This neural stimulation can be applied via non-penetrating spiral nerve cuff electrodes, though minimal information has been published regarding their long-term performance for multiple years after implantation.

**Methods:**

Since 2005, 14 human volunteers with cervical or thoracic spinal cord injuries, or upper limb amputation, were chronically implanted with a total of 50 spiral nerve cuff electrodes on 10 different nerves (mean time post-implant 6.7 ± 3.1 years). The primary outcome measures utilized in this study were muscle recruitment curves, charge thresholds, and percent overlap of recruited motor unit populations.

**Results:**

In the eight recipients still actively involved in research studies, 44/45 of the spiral contacts were still functional. In four participants regularly studied over the course of 1 month to 10.4 years, the charge thresholds of the majority of individual contacts remained stable over time. The four participants with spiral cuffs on their femoral nerves were all able to generate sufficient moment to keep the knees locked during standing after 2–4.5 years. The dorsiflexion moment produced by all four fibular nerve cuffs in the active participants exceeded the value required to prevent foot drop, but no tibial nerve cuffs were able to meet the plantarflexion moment that occurs during push-off at a normal walking speed. The selectivity of two multi-contact spiral cuffs was examined and both were still highly selective for different motor unit populations for up to 6.3 years after implantation.

**Conclusions:**

The spiral nerve cuffs examined remain functional in motor and sensory neuroprostheses for 2–11 years after implantation. They exhibit stable charge thresholds, clinically relevant recruitment properties, and functional muscle selectivity. Non-penetrating spiral nerve cuff electrodes appear to be a suitable option for long-term clinical use on human peripheral nerves in implanted neuroprostheses.

## Background

When electrical stimulation is applied to intact peripheral motor nerves, it is possible to contract the muscles that they innervate. Such implantable motor system neuroprostheses are useful for people with spinal cord injury (SCI), stroke, multiple sclerosis, and cerebral palsy. This technique can restore a person’s ability to stand and walk [1–7] and to reach and grasp objects [8–11]. The restoration of these functional tasks leads to an increase in independence and quality of life. Motor neuroprostheses can also have physiological benefits, such as improvements in muscular strength [12–14], motor control [14], lean muscle mass [15], motor and sensory ability [15], bone mineral density [16], and physiological responses after exercise tests [17, 18].

When stimulation is applied to intact sensory nerves, it is possible to evoke feelings of tactile sensation by exciting afferent nerve fibers. Sensory restoration via the stimulation of intact proximal sensory pathways in people with amputations is a useful tool for operating prostheses. Currently, prosthesis users rely on visual feedback, auditory feedback [19], and/or interactions between the residual limb and the prosthesis socket. The implantation of a permanent sensory restoration system could reduce phantom limb pain [20], enhance prosthesis embodiment [21], and improve performance of grasping tasks [22].

**Fig. 1:**
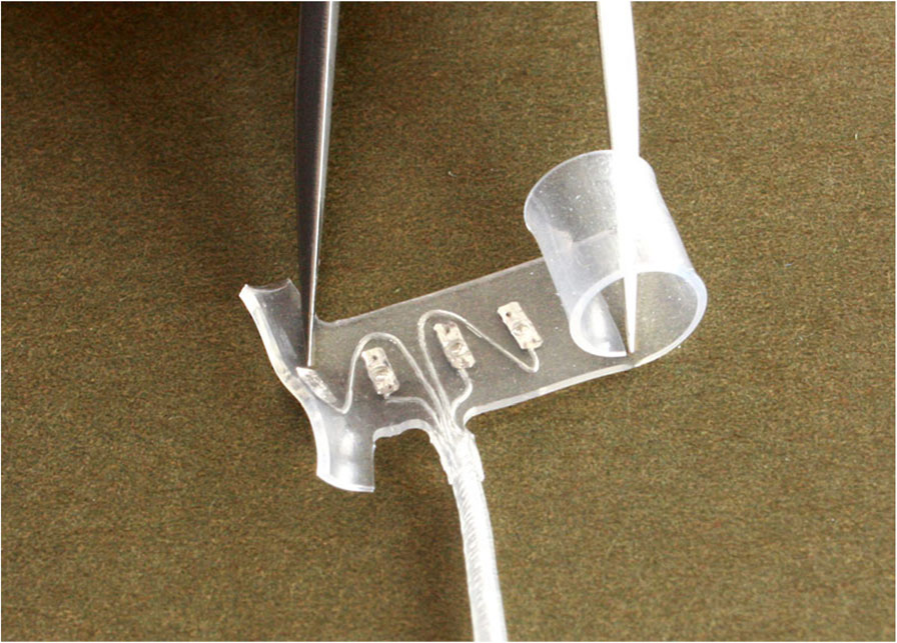
Standard spiral nerve cuff electrode. *Caption*: A standard self-sizing spiral nerve cuff electrode (d = 4 mm) licensed from Case Western Reserve University. In this study, 14 human participants were implanted with spiral cuffs on ten different nerves. Photo courtesy of Ardiem Medical, Inc. [48]

Neural stimulation can be applied via non-penetrating nerve cuff electrodes. Nerve cuff electrodes (NCEs) gently wrap around the outside of a peripheral nerve and do not breach the epineurium. One type of NCE is called a “spiral,” which consists of a self-curling polymer sheath that wraps around the nerve twice to create a circular transverse cross section [23] (Fig. 1). Spiral cuffs come in different nominal diameters, but are also capable of “self-sizing” to intermediate nerve sizes at implantation. The stimulation contacts within an appropriately sized spiral cuff are usually evenly spaced around the circumference of the nerve. They can also be secured on the nerve without the use of sutures. This allows the spiral cuff to do two things: first, it can stretch or open in order to maintain a snug fit with a nerve that is larger in diameter than the spiral. Secondly, when the spiral cuff is placed under strain, it can dislodge in order to prevent the transmission of force directly to the nerve [24].

A spiral cuff with multiple embedded stimulation contacts has the potential to isolate and activate multiple different fascicles within a nerve. They primarily access the fascicles that are closest to the epineurium. The central axon populations are difficult to activate without the simultaneous activation of nearby fascicles, unless techniques involving multiple current sources and sinks are used to steer the current between the contacts. If it is not necessary to access all fascicles within the nerve, spiral cuffs are a desirable choice.

Spiral cuffs have been successful in both animal and human applications. Spiral cuffs implanted on the cat sciatic nerve were able to selectively activate individual motor nerve fascicles in two acute studies [25, 26] and chronically in one 34-week study [27]. Spiral cuffs were then tested intraoperatively in the upper extremity nerves of 21 human participants [28]. It was determined that the mean stimulation charge thresholds were not significantly higher than the previous animal studies. Spiral cuff electrodes were then chronically implanted on the upper extremity nerves [11, 29–31], lower extremity nerves [4], and optic nerve of human participants [32]. For those paralyzed by SCI, the cuffs successfully restored select hand, arm, and leg motor functions. For those with amputations, a spiral cuff elicited tactile sensations referred to the missing limb of the user [30, 31]. The spiral cuff on the human optic nerve evoked distinct perceptions or “phosphenes” across the visual field.

Although spiral nerve cuff electrodes have been successful in both motor and sensory clinical applications, to our knowledge minimal information has been published regarding their long-term performance for multiple years after implantation. In this paper, we analyze the longitudinal behavior of stimulating spiral nerve cuff electrodes in 14 human recipients of implanted neuroprostheses, and focus specifically on cuff performance in four individuals who have been recipients of multi-contact spiral NCEs for over five and up to 11 years.

## Methods

### Subject selection & implanted technology

Fourteen human volunteers were implanted with spiral nerve cuff electrodes for use in motor or sensory neuroprostheses at Case Western Reserve University (CWRU) since 2005 (Table 1). All spiral nerve cuff electrodes were fabricated with a single lead according to the same design standards, and constructed from identical materials. At time of implantation, all surgeons measured the diameter of the target nerves intraoperatively and selected spiral cuffs of a corresponding size (2–10 mm in diameter) from an inventory of sterile devices.

**Table 1:** Summary of spiral nerve cuff electrode recipients and observations. *Caption:* A summary of the 14 spiral nerve cuff electrode recipients. Information about their type of spiral cuff, location of the cuff, implant date, observations regarding performance, and the date of last follow-up are presented. For participants with SCI, the injury level and Abbreviated Injury Scale (AIS) classification is given

Subject	Injury	Type of Cuff	Location of Cuff	Date of Implant	Observations	Date of Last Follow-Up
01	Brown-Séquard syndrome	Two 4-contact spirals, four monopolar spirals	Radial, Musculocutaneous, Suprascapular, Axillary, Long Thoracic, Thoracodorsal	11/08/2005	All contacts operational.	8/2016
02	T6 AIS A	Two 4-contact spirals	Femoral	12/09/2005	All contacts operational.	10/2014
03	C5 AIS A	Two 4-contact spirals, three monopolar spirals	Radial, Suprascapular, Long Thoracic, Thoracodorsal (bilateral)	7/25/2006	One monopolar cuff pulled off the right thoracodorsal nerve (between 9/8/2006 and 10/24/2006). All other contacts operational.	9/2013
04	C5 AIS B	Two 4-contact spirals	Femoral	9/19/2006	All contacts operational.	8/2014
05	C3 AIS A	Four 4-contact spirals, two monopolar spirals	Radial, Musculocutaneous, Suprascapular, Axillary, Long Thoracic, Thoracodorsal	3/25/2008	All contacts operational.	3/2014
06	C6 AIS D	Two monopolar spirals	Fibular	8/13/2010	All contacts operational.	12/2015
07	C7 AIS B	Two 4-contact spirals	Femoral	8/30/2010	All contacts operational.	1/2017
08	T11 AIS B	Two 4-contact spirals	Femoral	5/2/2011	One contact does not elicit a response. The other seven contacts are operational.	9/2016
09	T4 AIS B	Six monopolar spirals	Femoral, tibial, fibular	1/16/2012	All contacts operational.	7/2016
10	T6 AIS B	Six monopolar spirals	Femoral, tibial, fibular	5/15/2012	All contacts operational.	10/2016
11	Trans-radial amputation	One 4-contact spiral	Radial	5/24/2012	All contacts operational.	1/2017
12	T4 AIS A	Six monopolar spirals	Femoral, tibial, fibular	12/11/2012	Cuffs intact but unobservable due to stimulator explant (3/2013).	3/2013
13	T4 AIS A	Two monopolar spirals	Femoral	11/20/2014	All cuffs operational.	11/2016
14	T3 AIS A	Two monopolar spirals	Femoral	12/2/2014	All cuffs operational.	1/2017

Thirteen of these volunteers were paralyzed by SCI and received upper or lower extremity motor system neuroprostheses. Subjects 01, 03, and 05 have reaching and grasping neuroprostheses; Subjects 02, 04, 07, 08, 13, and 14 have standing neuroprostheses; Subject 06 has a stepping neuroprosthesis; and Subjects 09 and 10 have standing and stepping neuroprostheses. Subject 11 has an upper limb amputation and uses the system for somatosensory feedback of interactions between his end effector and the environment. The performance of the spiral cuffs in Subject 12 is unobservable due to explantation of the pulse generator. Specific details of the electrode placement have been described elsewhere [6, 7, 11, 30, 33–35]. The Institutional Review Boards (IRB) of the MetroHealth Medical Center and/or the Louis Stokes Cleveland Department of Veterans Affairs Medical Center approved each study and the subjects provided consent prior to participation. All studies were conducted under a Food and Drug Administration (FDA) Investigational Device Exemption.

Each cuff electrode was connected to an implanted stimulator–telemeter (IST) developed at Case Western Reserve University [36, 37]. An IST is capable of measuring sensor signals, telemetering the data for signal processing, and receiving power and control information from an external control unit. For the lower extremity motor system users, the control unit communicates with the IST via inductive radiofrequency coils. The user places an external coil on his or her skin directly over the inductive coil that sits within the implanted stimulator. Once the coil is placed, they can send stimulation commands by pressing pre-programmed buttons on the external control unit. For the upper extremity motor system users, EMG signals from implanted electrodes are telemetered out of the body through the coil. The external control unit processes these control signals and appropriate stimulation commands are sent back through the coil to the IST.

The IST can deliver constant-current, charge-balanced biphasic stimulus pulses with pulse widths between 1 and 255 μs and pulse amplitudes between 0.1–20 mA. The titanium case of the IST serves as the common return electrode. For cuffs with multiple embedded contacts, each contact was separately connected to its own independent stimulation channel. For monopolar cuff electrodes where all four contacts were connected in series, only one variable stimulation channel was used. Stimulating current was generally limited to 2.1 mA or less, and the pulse widths were adjusted to maintain safe charge density limits [38]. The stimulation frequency used for the upper extremity motor subjects was 12.5 Hz. The stimulation frequency used when calculating charge threshold for Subject 11 was 20 Hz for the first 2.2 years and 100 Hz for the remaining data sets. The frequency changed in order to match the value typically used in functional testing. A frequency of 20 Hz was used with the remaining participants. Pulse amplitude was varied between 0.3-18 mA (Table 2).

**Table 2:** Summary of the outcome measures and stimulation parameters for active research participants. *Caption:* An overview of the implant date, outcome measures, and stimulation pulse amplitude used for the subjects focused on in this study. The stimulation frequency was 12.5 Hz for Subject 01, 20–100 Hz Subject 11, and 20 Hz for the remaining participants. Pulse width was varied between 1 and 255 μs

Subject	Implant Date	Outcome Measures	Pulse Amplitude
S01	11/8/2005	8 charge threshold sessions from one month to 10.4 years post-implantation.	0.8 mA in 58% of the trials; 1.4 mA in 24% of the trials; 2.1 mA in 18% of the trials
S07	8/30/2010	10 charge threshold sessions from one month to 5.8 years post-implantation; measured moments to analyze muscle selectivity at 6.3 years post-implantation	1.4 mA
S08	5/2/2011	11 charge threshold sessions from one month to 5.3 years post-implantation; measured moments to analyze muscle selectivity at 4.2 years post-implantation	0.8 mA
S09	1/16/2012	6 recruitment curves at 4.5 years post-implantation	Left femoral: 2.1 mA Right femoral: 18 mA Left tibial: 2.1 mA Right tibial: 1.4 mA Left fibular: 1.4 mA Right fibular: 20 mA
S10	5/15/2012	6 recruitment curves at 4.5 years post-implantation	Left femoral: 1.4 mA Right femoral: 0.8 mA Right tibial: 2.1 mA Right fibular: 2.1 mA Left tibial: 1.4 mA Left fibular: 2.1 mA
S11	5/24/2012	13 charge threshold sessions from three weeks to 4.6 years post-implantation.	Radial contact #1: 0.5–0.7 mA Radial contact #2: 0.4–0.7 mA Radial contact #3: 0.3–0.6 mA Radial contact #4: 0.3–0.5 mA
S13	11/20/2014	2 recruitment curves at 2 years post-implantation	1.5 mA
S14	12/2/2014	2 recruitment curves at 2 years post-implantation	Left femoral: 7 mA Right femoral: 8 mA

### Data collection

Moments about the knee and ankle joints were measured with a 6-DOF load cell (JR3, Woodland, CA, USA) on a Biodex dynamometer. Knee extension moment was measured in response to varying the stimulation delivered by spiral cuffs on the femoral nerve for six volunteers with low tetraplegia and paraplegia (Subjects 07–10, 13, 14). The knee joint was fixed at 20° of flexion and its center of rotation was aligned with one axis of the load cell. For Subjects 09 and 10, ankle moments were measured in response to varying the stimulation delivered via tibial and fibular spiral cuffs. The ankle was fixed at 0° with the load cell centered about the knee joint. The resulting moment data were sampled at 150 Hz, low-pass filtered at 31.25 Hz, and normalized to body weight.

Pulse width-modulated moment recruitment curves were generated for every independent contact. Joint moment was measured as pulse widths were varied between 0 and 255 μs. For each participant, the lowest pulse amplitude that could achieve full recruitment of the targeted motor population was selected. Pulse width and amplitude were limited such that the charge stayed within safe limits [38].

### Outcome measures

**Fig. 2:**
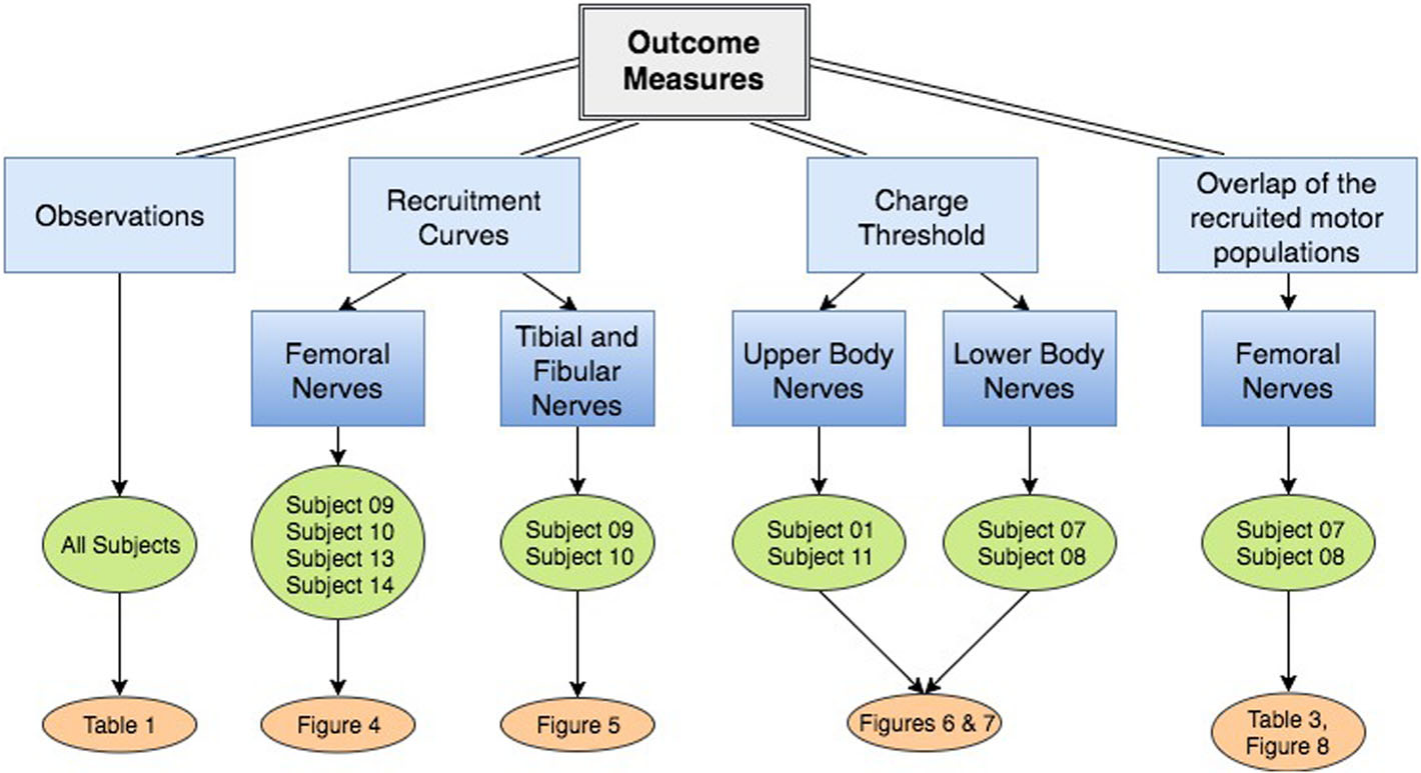
Flow chart of the outcome measures used in this study. *Caption*: An overview of the outcome measures and the corresponding research participants

The primary outcome measures utilized to assess chronic spiral nerve cuff performance were recruitment curves, charge threshold, and percent overlap of the recruited motor unit populations (Fig. 2). When collecting recruitment curves, pulse trains were used to generate “tetanic” moments about the knee and ankle joints. We present the recruitment curves generated in one session that occurred 4.5 years after implantation for Subjects 09 and 10, and 2 years after implantation for Subjects 13 and 14. Femoral nerve recruitment curves are presented for Subjects 09, 10, 13, and 14, and tibial and fibular nerve recruitment curves are presented for just Subjects 09 and 10.

**Fig. 3:**
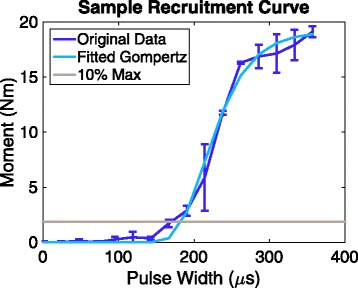
Example of a recruitment curve. *Caption*: Gompertz model fitted to a pulse width-modulated recruitment curve. The horizontal line denotes 10% of the maximum moment. The pulse width corresponding to the 10% maximum moment was multiplied by the pulse amplitude and used as the “charge threshold” for Subjects 07 and 08

Charge threshold was determined in three ways due to the subjects’ original participation in different studies. For Subject 01, charge threshold was defined as the minimum charge needed to evoke a visible muscle contraction of the upper extremity musculature. Since the number of channels on the implanted stimulator was limited, one radial nerve cuff channel and two musculocutaneous cuff channels were not connected and those thresholds were not measured. Charge thresholds for Subject 01 were repeatedly measured in eight sessions spanning 10.4 years. To determine charge thresholds for Subjects 07 and 08, Gompertz models were fit to the recruitment curves [35]. We set the charge threshold as the charge (pulse amplitude * pulse width) required to obtain 10% of the maximum moment produced, as estimated by the Gompertz model (Fig. 3). Over a period of 5+ years after implantation, we conducted ten sessions for measuring charge threshold in Subject 07 and 11 sessions for Subject 08. For Subject 11, charge threshold was determined by performing an unbiased adaptive procedure called “single-interval adjustment matrix” (SIAM) [30, 39, 40]. The subject was asked whether or not he felt a stimulation-induced sensation, and the stimulation parameters were adjusted based on his response. Stimulation parameters were set for a target performance of 50% and true stimulation was provided 50% of the time. The threshold search continued until there were approximately 12–16 “reversals” between when sensation was or was not perceived. Charge thresholds for Subject 11 were repeatedly measured in 13 sessions spanning 4.6 years. For each electrode contact in the four subjects, we evaluated the relationship between time and charge threshold by generating a Pearson’s correlation coefficient using MATLAB’s “corrcoef” function.

To assess whether multi-contact cuffs remain selective for independent populations of motor units for multiple years post-implantation, we measured the percent overlap of the recruited motor unit populations [35]. We first measured the tetanic moment produced by each individual contact (M_i_, M_j_…). We then measured the tetanic moment when two contacts were stimulated within 2 ms of each other (*M*_*i* ∪ *j*_), while the units recruited by the first contact were still in their refractory periods when the second pulse was delivered. If two contacts were completely independent and did not have any overlap in the motor unit populations they activated, their individual moments added linearly when the contacts were stimulated 2 ms apart. If there was some overlap, their combined moment was less than the sum of their individual moments because some of the motor units were still in their refractory period and would not respond to additional stimulation. Percent overlap (O_i,j_) was defined as:

$$ {O}_{i, j}={100}^{\ast}\left[\frac{M_i+{M}_j-{M}_{i\cup j}}{M_{i\cup j}}\right] $$

The lower the percent overlap (O_i,j_), the more selective the spiral cuff was in recruiting independent motor unit populations. For this analysis, we focused on Subject 07’s right femoral cuff during one session that occurred 6.3 years after implantation, and Subject 08’s left femoral cuff during a session that occurred after 4.2 years. Only two cuffs were tested due to time constraints. The pulse widths for each channel were chosen so that they generated approximately equal moments. For Subject 07, pulse amplitude was set to 1.4 mA and the pulse widths were 82 μs for Channel 1, 100 μs for Channel 2, 95 μs for Channel 3, and 143 μs for Channel 4. For Subject 08, amplitude was set to 0.8 mA and the pulse widths were 114 μs for Channel 1, 89 μs for Channel 2, and 73 μs for Channel 4.

## Results

### Observations about spiral cuff electrode performance

The 13 SCI participants in this study varied by injury level (C3-T11), nerve cuff location (various upper and lower extremity nerves), and the number of independent stimulation contacts (one to four) per cuff (Table 1). One individual with trans-radial limb loss was included and implanted with a four-contact spiral cuff on his radial nerve. Across all 14 recipients, the aggregate experience amounts to more than 349 cuff-years. The performances of the spiral cuffs implanted in Subjects 01, 07–11, 13, and 14 were verified within the last year (referred to as the eight “active participants” in ongoing research studies). Subject 03 was last tested in 2013; Subjects 02, 04, and 05 were last tested in 2014; and Subject 06 was last tested in 2015. The implanted stimulator was explanted from Subject 12 in 2013 due to a perioperative infection that did not involve the spiral nerve cuffs, and the contacts can no longer be tested. Out of the eight active participants, there are 27 cuffs that have a total of 45 independent contacts. One contact within a multi-contact spiral cuff in Subject 08 does not evoke a motor or sensory response and therefore may not be near excitable nerve fibers. Forty-four out of 45 contacts (98%) were reported as operational and evoking a motor or sensory response in the active research participants.

The six remaining participants no longer participate in research studies. They were implanted with 23 spiral cuffs with a total of 56 independent contacts. One monopolar spiral cuff in Subject 03 pulled off of the right thoracodorsal nerve. This could be due to reports of the subject hooking his right arm around the handles of his wheelchair; this could have applied tension to the lead, since a lack of redundant lead length for strain relief was noted during surgery. As previously mentioned, the stimulator in Subject 12 was explanted after 3 months; all electrode contacts were tested and operational at that time. His nerve cuff electrodes remain intact and have caused no clinical issues, although they are inaccessible to stimulation. No other adverse events related to the operation of the spiral cuff electrodes have been reported.

### Recruitment curves

**Fig. 4:**
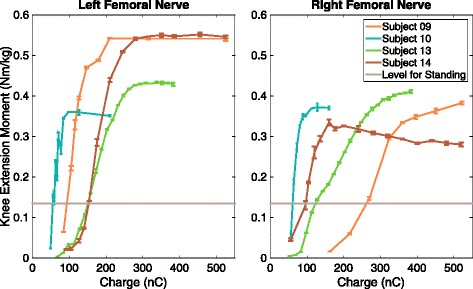
Recruitment curves for the femoral nerve cuff electrodes in Subjects 09, 10, 13, 14. *Caption*: Pulse width-modulated recruitment curves normalized to body weight for four subjects with monopolar spiral nerve cuff electrodes on their femoral nerves. The positive moment values represent knee extension. When the data were collected, Subjects 09 and 10 had been implanted for 4.5 years, and Subjects 13 and 14 had been implanted for 2 years

**Fig. 5:**
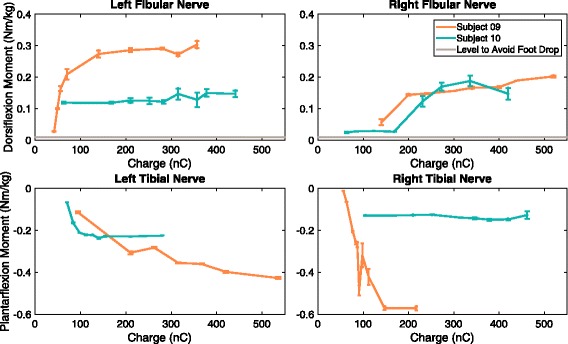
Recruitment curves for the fibular and tibial nerve cuff electrodes in Subjects 09 and 10. *Caption*: Pulse width-modulated recruitment curves normalized to body weight for two subjects with monopolar spiral nerve cuff electrodes on their tibial and fibular nerves. Negative moment values represent plantarflexion; positive moment values are dorsiflexion. 0.01 Nm/kg is normally required to prevent foot drop [42] and is represented by a horizontal gray line. Plantarflexion moment during push-off at a normal walking speed is 1.6 Nm/kg [43], which was too large to be shown. When the data were collected, Subjects 09 and 10 had been implanted for 4.5 years

Within the past 6 months, pulse width-modulated recruitment curves were generated for four subjects with monopolar spiral nerve cuff electrodes on their femoral nerves (Fig. 4). The knee extension moments produced by all eight cuffs exceeded the 0.135 Nm/kg required to keep the knees locked during standing [41], demonstrating that the devices remain functional for at least 2–4.5 years after implantation. Figure 5 shows the most recent recruitment curves gathered for two subjects (09 & 10) with monopolar nerve cuff electrodes implanted on the tibial and fibular nerves below the knee. All four monopolar fibular nerve cuffs were able to produce a dorsiflexion moment greater than 0.01 Nm/kg, the level needed to prevent foot drop [42]. Plantarflexion moment during push-off at a normal walking speed is 1.6 Nm/kg [43], but the moments produced by the tibial nerve cuffs did not reach this value. When stimulating Subject 10’s left fibular and right tibial nerve cuffs, knee flexion and reflexive responses also occurred, which could influence the moment measured about the ankle. Subject 10 did not regularly use her system, so it is possible that her muscles deconditioned.

### Charge thresholds up to 10.4 years

**Fig. 6:**
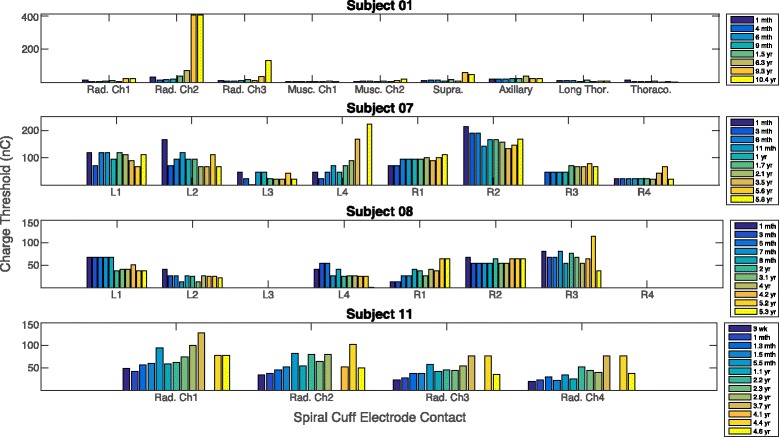
Charge thresholds measured in Subjects 01, 07, 08, and 11 over multiple years. *Caption*: Charge thresholds were measured over a period of 10.4 years for Subject 01, 5.8 years for Subject 07, 5.3 years for Subject 08, and 4.6 years for Subject 11. “Rad” = radial nerve, “Musc” = musculocutaneous nerve, “Supra” = suprascapular nerve, “Long Thor” = long thoracic nerve, “Thoraco” = thoracodorsal nerve. “L1” represents the first contact on the multi-contact left femoral spiral nerve cuff electrode

Charge thresholds remained stable in the majority of contacts within the 11 spiral nerve cuff electrodes in the four subjects who received the implanted devices between 5 and 11 years ago (Fig. 6). The acute stabilization in the months directly after implantation is discussed in more detail elsewhere for these participants [4, 29, 33, 39]. Subject 01 has two multi-contact cuffs and four monopolar cuffs; Subjects 07 and 08 have multi-contact spiral nerve cuff electrodes on their bilateral femoral nerves; Subject 11 has one multi-contact cuff on his radial nerve. Two contacts in Subject 08 are not included because they did not elicit discernable motor responses. Of the 27 contacts, only eight had a significant increase in charge threshold over time (linear regression, *p* < 0.05). The charge threshold of the second contact on Subject 01’s multi-contact radial nerve cuff (Rad. Ch2) increased by 370.9nC between the first and last sessions. Rad. Ch3 increased by 120.3nC, Musc. Ch2 increased by 14.4nC, and the suprascapular cuff increased by 36.2nC. For Subject 07, the fourth contact in the left femoral cuff (L4) increased by 175nC, R1 increased by 40.6nC, and R3 increased by 67.2nC. The first contact on Subject 08’s right femoral cuff (R1) increased by 50.4nC. These gradual increases over time or increases during the two most recent sessions likely indicate a permanent change in charge threshold. Even with these increases in stimulation parameters, the stimulation levels were within safe limits. The non-significant fluctuations in charge threshold over time, for example the increase in Subject 08’s R3 threshold at the 5.2-year session and decrease at 5.3 years, could be a result of leg posture during measurement. Even slight differences in the knee angle could affect the measured moment.

**Fig. 7:**
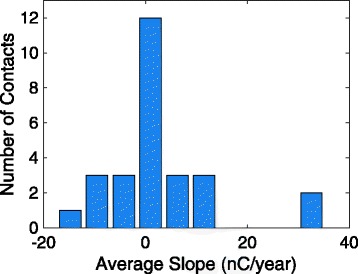
Average change in charge threshold per year for Subjects 01, 07, 08, and 11. *Caption*: The average change in charge threshold that each contact in a spiral nerve cuff electrode underwent each year post-implantation. There were 27 contacts in total: four from monopolar cuffs in Subject 01 and 23 from multi-contact cuffs in Subjects 01, 07, 08, and 11. The total time span was 10.4 years for Subject 01, 5.8 years for Subject 07, 5.3 years for Subject 08, and 4.6 years for Subject 11

Figure 7 shows each contact’s average change in charge threshold per year. The majority of contacts show excellent stability with average changes in threshold close to 0nC/year, with 20 out of the 27 contacts (74%) exhibiting less than a 5nC average increase in charge threshold per year.

### Percent overlap of recruited motor unit populations

**Fig. 8:**
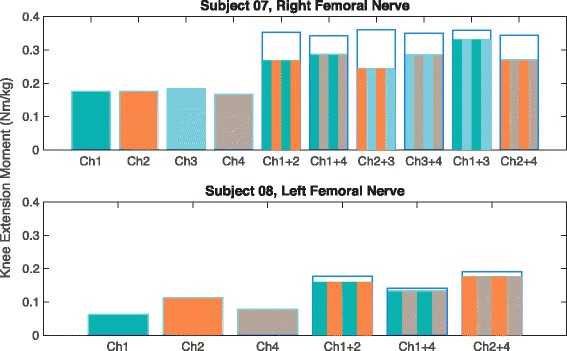
Individual and combined tetanic moments for the contacts in Subject 07’s and 08’s multi-contact spiral nerve cuff electrodes. *Caption*: Individual and combined tetanic moments for the contacts in Subject 07’s right femoral multi-contact cuff 6.3 years after implantation and Subject 08’s left femoral cuff after 4.2 years. Contacts were stimulated individually and pairs of contacts were stimulated within 2 ms of each other. If the two contacts activate completely independent motor units, their individual moments would add linearly, which is represented by the empty bars with blue outlines. The striped bars represent the additive moment that was measured experimentally

Multi-contact cuffs on the femoral nerves of Subject 07 and 08 were still selective for different motor populations after being implanted for several years (Table 3, Fig. 8). Stimulating currents were applied to contacts individually and in pairs within 2 ms of each other. The percent overlap (O_i,j_) ranged from 5.5% to 47.4%, with an average overlap of 20.2%. The contacts on opposite sides of the nerve (1&3, 2&4) had 14.6% overlap on average. The contacts on adjacent sides of the nerve (1&2, 1&4, 2&3, 3&4) had 23% overlap on average, which was expected due to their closer proximity. Contacts 1 and 4 in Subject 08 were on adjacent sides of the femoral nerve yet they had the least amount of overlap (5.5%). While there was some overlap in the recruited motor populations after 4.2 years post-implantation, the resulting moment was 0.134 Nm/kg, which is approximately equal to the 0.135 Nm/kg needed to stand. It is possible that these contacts had the least amount of overlap because neither contact generated a strong enough motor response to spill over to another motor unit population. The two contacts with the second smallest percent overlap were Contacts 1 and 3 on opposite sides of Subject 07’s right femoral nerve cuff. When the two contacts were stimulated within 2 ms, the resulting moment was 0.33 Nm/kg, which easily exceeds the 0.135 Nm/kg needed to stand. In 2013, Fisher et al. performed overlap tests with these same two participants [35]. In that study, our Subject 07 is their Subject 1 and our Subject 08 is their Subject 2. There are four pairs of contacts that are presented in both this and the Fisher et al. study. For Subject 07, they measured the overlap of one contact pair, Right 2&4, during four sessions over 27–53 weeks after implantation. After 6.3 years post-implantation, the measured overlap was greater than the original standard deviation range. The percent overlap we measured was 26.8%, which is close to the 25% overlap needed to best prevent knee buckling [44]. For Subject 08, they measured the overlap for three contact pairs during three sessions over 14–37 weeks after implantation. After 4.2 years, two out of those three pairs still fell within the standard deviation range. The pair that fell outside of this range, Left 1&2, had 11.3% overlap. Percent overlap is below 25%, so this contact pair is still highly functional in preventing knee buckling. These results demonstrate that motor unit selectivity is maintained for up to 6.3 years after implantation.

**Table 3:** Percent overlap values for contact pairs within two spiral nerve cuff electrodes. *Caption:* The percent overlap of recruited motor unit populations for pairs of contacts within spiral cuffs on the femoral nerves of Subjects 07 and 08. Overlap was calculated after 6.3 years for Subject 07 and after 4.2 years for Subject 08. An overlap value of 0% indicates that the two contacts activate completely independent motor units

Subject	Contacts	Overlap
S07	R1 & R2	31.9%
	R1 & R4	19.6%
	R2 & R3	47.4%
	R3 & R4	22.4%
	R1 & R3	8.5%
	R2 & R4	26.8%
S08	L1 & L2	11.3%
	L1 & L4	5.5%
	L2 & L4	8.6%

## Discussion

The recruitment curves, charge thresholds, and overlap in recruited motor unit populations of 50 spiral nerve cuff electrodes implanted across 14 subjects and 10 different nerves in the upper and lower extremities over the past 11 years were analyzed. Of this six participants not actively involved in research studies, only one spiral cuff was unable to activate its neural target. When this recipient moved his arm into extreme positions, excessive strain was placed on the electrode-to-IST lead. Under strain, the spiral cuff is designed to dislodge in order to prevent the transmission of force directly to the nerve [24]. This participant no longer uses his neuroprosthesis for hand motor function. This is mainly due to pre-existing partial ulnar nerve denervation, which limited the ability of the neuroprosthesis to restore hand opening and lateral grasp force; it was not due to the performance of the spiral nerve cuffs. Of the eight active participants, one independent contact within one spiral cuff electrode did not elicit a sensory or motor response, and may not be located near excitable nerve fibers. Another contact within a spiral cuff evoked sensations of pain rather than the motor response intended for the motor neuroprosthesis. It was likely located closer to the sensory fascicles than the motor fascicles within the femoral nerve, and so, was still considered operational and effective at exciting neural tissue. Ninety-eight percent of the independent contacts were reported as operational and evoking a motor or sensory response in the active participants.

The recruitment curve and percent overlap experiments were not performed directly after implantation or in a longitudinal fashion over multiple years. However, the results demonstrated that after multiple years, spiral cuffs are capable of recruiting the targeted motor population. The four participants with monopolar spiral cuffs on their femoral nerves were all able to generate enough moment to keep the knees locked during standing. The dorsiflexion moment produced by all four fibular spiral cuffs also exceeded the value required to prevent foot drop. For both these analyses, while there could have been a decrease in the motor response over time, it does not hinder the functionality of the neuroprostheses. The plantarflexion moments produced by the tibial nerve cuffs in Subjects 09 and 10 did not meet the value that occurs during push-off at a normal walking speed. This could be due to a lack of exercise, the location of the implanted spiral nerve cuff with respect to nerve branching, or the visual approximation of knee and joint angles during the experimental set-up. It is also possible that the moment was stronger immediately after implantation but was not measured. Subject 10 stopped using her system at home and did not regularly participate in research studies, so it is possible that her muscles became deconditioned. Additionally, walking speed with neural stimulation is reported to be slower than normal [45, 46] and therefore strong plantarflexion may not be essential in today’s lower extremity neural prostheses.

For the four participants studied at regular intervals after implantation, most charge thresholds remained stable over time. Eight out of the 27 contacts demonstrated an upward trend in the charge thresholds necessary to evoke a response, though the levels were still within safe levels of stimulation. The jump in charge threshold for the second contact in Subject 01’s radial nerve cuff may also be misleading. The pulse amplitude levels of the implanted stimulator jump from 2.1 mA to 18 mA, so it is possible that the true charge threshold lies between them. Overall, the charge threshold analysis demonstrates that the spiral cuffs maintained a close proximity to the nerve and remained physically intact and did not affect nerve function for up to 10.4 years.

Two multi-contact spiral cuffs in Subjects 07 and 08 were reported as selective for different motor populations by Fisher et al. in 2013 [35], and in this study these same electrodes still demonstrated motor unit selectivity. The contacts within a spiral cuff did recruit some overlapping motor unit populations. However, their recruited regions were still independent enough that the moment required to keep the knees locked during standing could be achieved when stimulation was applied via just two out of the four contacts.

The stimulation frequency used for most participants was under 50 Hz for all but one participant. The suggested limits for charge density were typically calculated at 50 Hz [38], therefore the safety limits for a frequency of 100 Hz (as used in Subject 11) are not as clear. The amount of time that the stimulation is on is different for each participant. For example, Subject 01 has a neuroprosthesis used for hand function: stimulation can stay on for a few seconds during reaching and grasping motions, or for a few minutes while holding an object. Subjects 02, 04, 07, 08, 13 and 14 have neuroprostheses that can be used for standing. The stimulation remains on for as long as participants are able to stand, which can be up to 13 min [33] depending on their muscle strength. All participants except for Subject 11 can use their systems at home and their daily use varies [6]. The amount that the system is used at home will directly impact their long-term performance.

This study focused solely on the analysis of chronically implanted spiral nerve cuff electrodes in humans. It would also be valuable to analyze data from other types of non-penetrating nerve cuff electrodes, such as the FINE (flat interface nerve electrode) [47]. The FINEs have more individual contacts with a smaller diameter and offer a higher degree of muscle selectivity than spiral cuffs. However, as a result, the contacts have higher current densities and require a more advanced implanted stimulator–telemeter. There is a tradeoff between selectivity and the cost and power of the implanted technology. When choosing a neural interface, it is important to consider both the long-term stability and the desired functional outcomes.

## Conclusion

For up to 11 years post-implant, non-penetrating spiral nerve cuff electrodes remain operational and functionally reliable with stable stimulation charge thresholds, joint moment-generating capacities, and overall performance in motor and sensory neuroprostheses. The spiral cuff electrode component of an implanted neuroprosthesis is robust, selective, free of serious adverse events, and free of medical complications. Spiral nerve cuff electrodes can be considered as a reliable peripheral nerve interface technology for long-term clinical use in implanted neuroprostheses.

## Abbreviations

AIS: Abbreviated Injury Scale
CWRU: Case Western Reserve University
FDA: Food and Drug Administration
FINE: flat interface nerve electrode
IRB: Institutional Review Board
L1: Is the first contact/channel on a subject’s left femoral nerve spiral cuff electrode
L2: Is the second contact/channel on a subject’s left femoral nerve spiral cuff electrode
L3: Is the third contact/channel on a subject’s left femoral nerve spiral cuff electrode
L4: Is the fourth contact/channel on a subject’s left femoral nerve spiral cuff electrode
Long Thor: Monopolar long thoracic nerve spiral cuff electrode
Musc. Ch1: First channel/contact of the musculocutaneous nerve spiral cuff electrode
Musc. Ch2: Second channel/contact of the musculocutaneous nerve spiral cuff electrode
NEC: Nerve cuff electrode
R1: Is the first contact/channel on a subject’s right femoral nerve spiral cuff electrode
R2: Is the second contact/channel on a subject’s right femoral nerve spiral cuff electrode
R3: Is the third contact/channel on a subject’s right femoral nerve spiral cuff electrode
R4: Is the fourth contact/channel on a subject’s right femoral nerve spiral cuff electrode
Rad. Ch2: Second channel/contact of the radial nerve spiral cuff electrode
Rad. Ch3: Third channel/contact of the radial nerve spiral cuff electrode
Rad: Rad. Ch: First channel/contact of the radial nerve spiral cuff electrode
SCI: Spinal cord injury
Supra: Monopolar suprascapular nerve spiral cuff electrode
Thoraco: Monopolar thoracodorsal nerve spiral cuff electrode
